# An experiential service-learning project on oral health examination and education

**DOI:** 10.1186/s12909-023-05020-7

**Published:** 2024-01-05

**Authors:** Liangyue Pang, Yan Zhou, Ye Tao, Lixia Yu, Yina Cao, Huancai Lin, Qinghui Zhi

**Affiliations:** 1 Hospital of Stomatology, Sun Yat-sen University, Guangzhou, Guangdong China; 2 Guangdong Provincial Key Laboratory of Stomatology, Sun Yat-sen University, Guangzhou, Guangdong China

**Keywords:** Field work, Oral health examination, Oral public health, Oral health education, Service-learning

## Abstract

**Background:**

It has been demonstrated that experiential service-learning is effective in fields including public health and medicine. Preventive Dentistry is a practical course, and Oral Health Examination and Education is a topic that is suitable for teaching with experiential service-learning. This study describes an example of experiential service-learning in Preventive Dentistry named “Oral Health Examination and Education Project” and also evaluates its effectiveness among dental students.

**Methods:**

A total of 108 dental students in their fourth year participated in this project in 2022. The project was composed of six sections: theoretical teaching, field investigation, data collection and analysis, investigation report writing and creating oral health education materials, oral health education and students’ evaluation of the project.

**Results:**

During this project, students learned how to perform surveys related to oral health, wrote an investigation report, created oral health education materials, and provided oral health education for children. Students were demonstrated an improvement in their academic performance for theoretical knowledge related to Oral Health Examination and Education in comparison with the students in the previous year. Over 90% of students expressed their preference for the learning method of experiential service and believed that it helped them to better understand the course material. They also recommended this teaching method for future classes.

**Conclusions:**

This study indicated that an experiential service-learning approach within this scope was highly beneficial to students because it provided them with the opportunity to understand the practical application of their coursework and obtain valuable experience in the field. This research suggests that oral epidemiology instructors in dental and oral public health programs should pay more attention to incorporate similar experiential projects into their curriculum with the aim of better preparing students for careers in oral public health.

## Introduction

Preventive Dentistry is one of the foundational courses of Stomatology education, which focuses on individual and group preventive health care strategies as well as the incidence of oral diseases in the general population [1]. It aims to improve the oral health level of the entire population with an emphasis on the expansion of services relate to oral medicine from simple in-hospital diagnosis and treatment to oral health care services at the community level outside the hospital, which has strong social practicality. One of the teaching topics for Preventive Dentistry is Oral Health Examination and Education. Traditionally, this section is taught through didactic lectures and group discussions, all of which can be effective for the introduction and explanation of relevant theoretical knowledge, such as epidemiologic concepts. However, the transition from theories in class to actual practice is not always successful [2], and students may encounter difficulty in grasping methodological concepts and their application to their future professions. Students also may have difficulty understanding the theory and concepts after taking these classes, and they may struggle in the process of applying this knowledge to real-world problems.

Studies have shown the effectiveness of field work and service-learning for the teaching of students in the fields of public health, medicine, and other disciplines related to health [2–3]. Experiential learning is a teaching method that encourages the application of the concepts and abilities learned in class to real-world public health situations [4]. It provides students with direct experience of the subject matter being studied rather than only allowing them to learn about it by reading or hearing the experiences of others. Providing students with earlier opportunities to participate in field work activities can enhance their learning outcomes [2]. This approach focuses on teaching through direct experience and reflection instead of solely transferring information from the instructor to the student. Service-learning is a type of education that integrates community service with academic instruction, enabling students to obtain practical experience while simultaneously satisfying the needs of their community [5]. It is considered an effective teaching method that can be employed in programs related to public health to provide students with a deeper comprehension of issues in the real world. This approach provides students with the ability to use their knowledge and skills to address community-based problems, which enhances their engagement and comprehension of the course material. The service-learning model can allow students to obtain an experiential and holistic understanding of the subject matter, which is of great value in preparing them for public health careers.

This article describes an experiential service-learning project that was carried out by dental students from Guanghua School of Stomatology, Sun Yat-sen University. This article provides an overview of the project and evaluates its effectiveness among dental students. This research also offers suggestions to instructors of preventive dentistry who are interested in the experiential service-learning approach.

## Methods

**Fig. 1:**
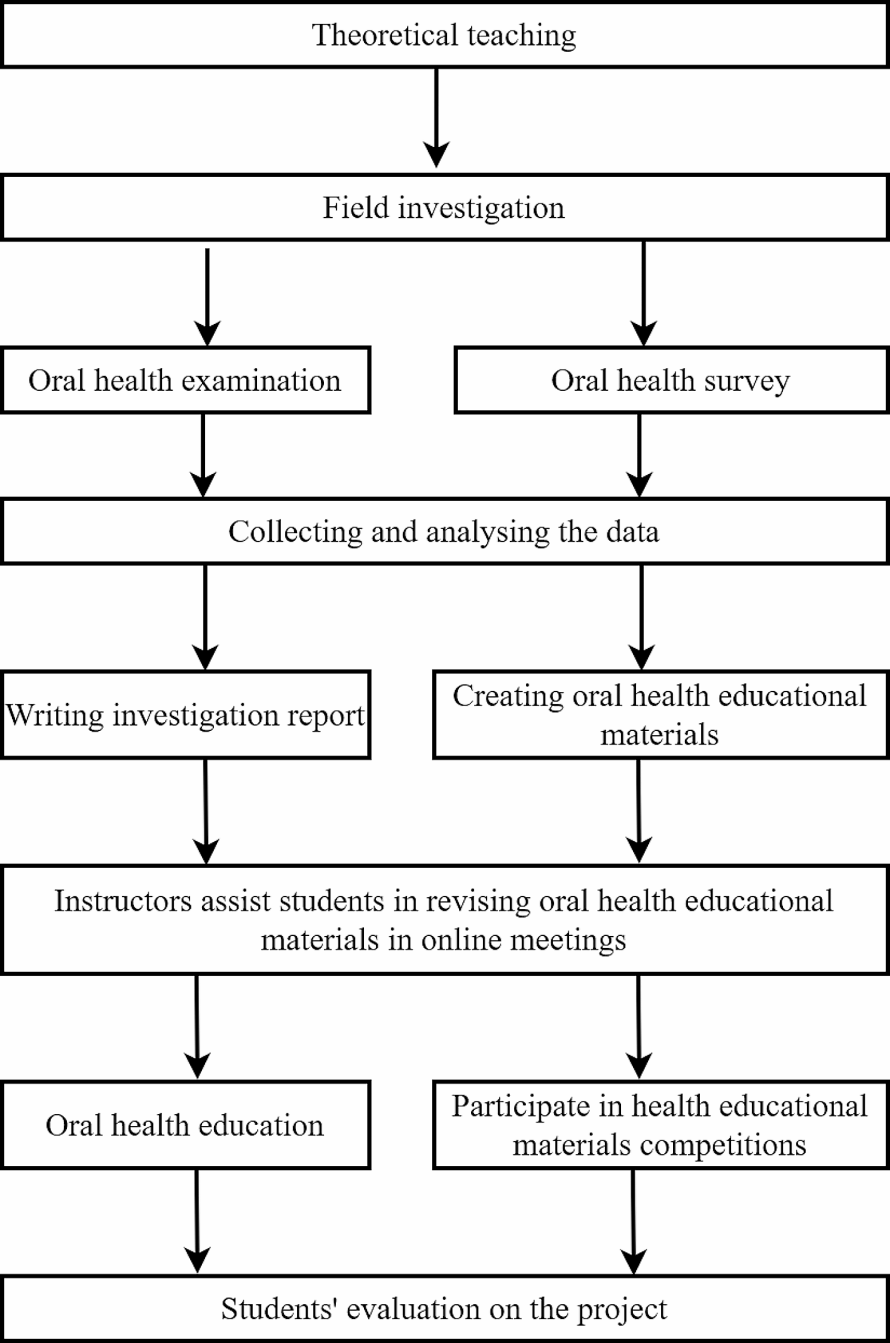
The flowchart of the project

A total of 108 dental students in their fourth year of dental education participated in a project titled Oral Health Examination and Education. The project was composed of six sections, including theoretical teaching, field investigation, data collection and analysis, investigation report writing and the creation of oral health education materials, provision of oral health education for children and students’ evaluation of the project. The flowchart of the project is displayed in Fig. 1, and the details are as follows. All methods were carried out in accordance with relevant guidelines and regulations in ethical declarations. The study was approved by the Medical Ethics Committee of the Hospital of Stomatology, Sun Yat-sen University (KQEC-2023-17-01). Informed consent was obtained from the participants or a parent and/or legal guardian.

### Theoretical teaching

The theoretical teaching section of the project was mainly completed by two associate professors and a lecturer of the preventive dentistry department through didactic lecture teaching on the concepts of Oral Health Examination and Education. The main topics were the concept of oral epidemiology, the techniques of oral health examination and an oral health knowledge survey, and the concepts and methods of oral health education.

### Field investigation

In this section, 108 dental students were taken by two lecturers to the kindergarten on the North Campus of Sun Yat-Sen University for the examination of oral health and oral health survey. The kindergarten on the North Campus of Sun Yat-sen University is composed of nine classes, three senior classes (children aged 5 to 6), three middle classes (children aged 4 to 5), and three junior classes (children aged 3 to 4), with 30 children in each class. A total of 108 dental students were divided into nine groups with 12 students in each group. The dental students in each group worked with one class of approximately 30 kindergarten students. The dental students conducted an oral health examination for each child and verbally administered an oral health knowledge survey for each child.

Before the oral examination, the instructor explained the standards for the oral examination and use of the interview questionnaire in detail. A five-year-old checklist from the Fourth National Oral Health Survey was adopted, and the examination of oral health was completed according to WHO standards [6]. During the oral examination, one student served as the examiner to check whether the children had caries while other students were in charge of documenting the children’s caries status (dmft). Uncertainties in the oral examination by the students were assessed by the instructors.

Prior to the service-learning experience at the Kindergarten, the instructor designed the oral health knowledge survey in Chinese. The survey included questions to assess children’ knowledge of oral health and their oral health behaviors. The questionnaire consisted of multiple-choice questions and yes/no questions. The questions were as follows: The time and frequency of teeth brushing; Which foods are harmful to teeth; Which habits harm oral health; Can sugar cause dental caries; Can I eat sweets after brushing my teeth at night; Can I avoid dental caries by sealing the pits and fissures; Is caries caused by bacteria on the teeth; and Can the occurrence of dental disease be prevented by yourself? There were 9 questions in total. One point was given to each correct answer, the highest score was 9 and the scores obtained by each child were recorded. During the oral health survey, a student who acted as an investigator asked the children for answers face to face. The investigator could only explain the meaning of the questions without inducing the children to answer them. Another student took notes according to the responses obtained from the children.

### Data collection and analysis

The dental students were required to collect checklists and questionnaires, enter the data into the EpiData database, and analyze the caries rate and questionnaire scores of the senior class, middle class, and junior class of kindergarten students.

### Investigation report writing and creation of oral health education materials for kindergarten students

Each group of students submitted a survey report based on the findings obtained from the data analysis. And then the dental students were asked to create oral health education materials based on the oral problems of kindergarten children in a month time. 4–5 students formed a creative team and they shared responsibility for developing a piece of material. The materials adopted for oral health education were PowerPoint presentations, posters, foldouts, movies, animations, and other formats. Through videoconferencing, instructors in the preventive dentistry department provided students with assistance in revising the oral health education materials.

### Provision of oral health education for children

The dental students went to the kindergarten on the North Campus of Sun Yat-Sen University accompanied by two instructors to conduct oral health education for the children in oral presentation, tale-telling and game play. During the process, the students provided one-on-one instruction to the children on brushing their teeth with tooth models. In addition, dental students were encouraged to participate in competitions related to the creation and dissemination of oral health education materials for college students held by the Chinese Stomatological Association.

### Students’ evaluation of the project

Tencent Questionnaire (https://wj.qq.com/) was used to collect students’ evaluations of the project, with the following specific questions and items: Do you prefer this experiential service-learning method in comparison with the traditional teaching method? Has the experiential service-learning method deepened the students’ understanding of the content in this course? Do you suggest this teaching method for future students? Please write an evaluation with suggestions and comments on the project related to the experiential service-learning.

## Results

The dental students analyzed the prevalence of caries, as well as the information about the awareness of knowledge and behaviors about oral health care in kindergarten children. The caries prevalence, mean dmft and the mean score of the questionnaire of 3 sub-groups were summarized in Table 1. The accuracy rate of each question in the questionnaire is shown in Table 2.

**Table 1:** The caries prevalence, mean dmft and the score of the questionnaire of 3 sub-groups

Classes	The caries prevalence	Mean dmft (Mean ± SD)	Score of the questionnaire (Median (IQR))
The senior classes	53.8%	1.75 ± 1.83	6 (5, 7)
The middle classes	47.1%	1.46 ± 1.52	5 (4, 6)
The junior classes	25.0%	1.02 ± 1.12	4 (3, 4)

**Table 2:** The accuracy rate of each question in the questionnaire

Question	The accuracy rate
The frequencies of teeth brushing	65.7%
The time of teeth brushing	41.6%
Which foods are harmful to teeth	49.3%
Which habits harm oral health	22.89%
Can I eat sweets after brushing my teeth at night?	94.58%
Is caries caused by bacteria on the teeth?	68.67%
Can sugar cause dental caries	91.56%
Can the occurrence of dental disease be prevented by yourself	74.69%
Can I avoid dental caries by sealing the pits and fissures?	10.84%

In accordance with the findings, the dental students created a report about oral examination. And they designed the oral health education plan based on the finding of the oral health examination and the oral health survey and created nine PowerPoint presentations and twelve popular oral health education materials. Among them, eight oral health education materials were part of a competition of college students’ works on the creation and dissemination of oral health education held by the Chinese Stomatological Association. One won first prize, two won second prize, and two won third prize.

In 2021, the dental students just attended lectures but did not participate in the service-learning experience, and the students in 2022 completed the experience that was described in this paper. Then, they both had a test of theoretical knowledge related to Oral Health Examination and Education. Therefore, to measure the impact of the service-learning experience, the scores were compared between these two grades students. But the maximum scores of that test were different in those two years. The maximum score in 2021 was 46 points and it was 56 points in 2022. As a result, the accuracy rate of this test was the only way to be comparable for those two grades students. The accuracy rate was the score of each student got from the test divided by the maximum score. Mann-Whitney U test was used to compare the accuracy rate of this test in 2022 and 2021 (Table 3). The result showed that the accuracy rate of 2022 was significantly higher than the 2021 cohort, suggesting that the experiential service-learning activity deepened dental students comprehension of concepts related to examination of children’ oral health and oral health education.

A questionnaire was adopted with the aim of collecting students’ evaluations of the project (Table 4). A total of 88 valid questionnaires were collected, with a response rate of 81.48%. Over 90% of students expressed their preference for the learning method of experiential service and were convinced that it helped them to better understand the course material. Students reported that the project was interesting and enjoyable in addition to reinforcing the content of the relevant course.

**Table 3:** Comparison of the theoretical knowledge accuracy rate in 2022 and 2021

	Median (IQR)		Mann-Whitney U test	
	2021	2022	Z	*p* value
Accuracy rate	0.80 (0.63, 0.87)	0.88 (0.80, 0.93)	5.26	<0.001

**Table 4:** Students’ evaluation of this project

Question	Yes (N)	Frequency (%)
Do you prefer this experiential service-learning method in comparison with the traditional teaching method?	83	94.32
Has the experiential service-learning method deepened the students’ understanding of the content in this course?	81	92.05
Do you suggest this teaching method for future students?	84	95.45

## Discussion

Experiential service-learning is a pedagogical approach that connects theory and practice by giving students the opportunity both to participate in a service that meets community needs and to reflect on the experience in class in order to gain a deeper understanding of the course content [7]. It is becoming increasingly popular and has been applied in many fields of higher education such as public health [2–3, 8], nursing education [9], engineering education [10], hospitality education [11] and so on. This project presents an example of the experiential teaching approach of service-learning in dental education. In fact, there were numerous service-learning applications in the field of dental hygiene [12–14], such as “Oral Health on Wheels” [12]. Different from other oral health education programs [12–14], this project integrated theoretical learning, dental epidemiological investigations, data analysis, creation of scientific oral health education materials and oral health education. Students used the data they collected to identify the oral problems encountered by children in kindergarten and their knowledge of oral health. They learned how to perform oral health surveys, provide oral health education, and apply the knowledge learned in the theoretical class to actual practice throughout the project. This program not only improved the students’ community service ability but also their ability to create scientific oral health education materials.

A previous study reported that experiential service-learning can result in substantial changes in students’ comprehension of specialized knowledge [7]. Similar to previous research, these findings also demonstrate that experiential service-learning enhances students’ comprehension of specialized knowledge and improves their theoretical performance. This phenomenon can be explained by two reasons. Firstly, service-learning engages students in active learning. They learned by participating in this project and reflecting on their experiences, which can be more effective than traditional methods such as lectures or readings [15]. Secondly, experiential service-learning provides students with the ability to apply their knowledge and skills to real-world situations and offers them the opportunity to serve their community and make a positive impact, which can be beneficial to their understanding of the relevance and practical application of their coursework in addition to providing a rewarding and meaningful experience.

Experiential service learning not only helps students develop a variety of professional and personal skills, including communication, problem solving, leadership, and teamwork that are of great value in various career and life contexts, but also enhances the faculty‒student relationship [8, 16–18]. Currently, the proportion of dentists and residents in China is approximately 1:8000 [19], which is far lower than the WHO-recommended approximately 1:5000 in developing countries [20], suggesting that China’s oral health resources are insufficient. Additional prevention measures are needed, and oral diseases prevention or oral health promotion work should be strengthened. Oral health education helps the public form good oral health habits, and oral examination can find problems early and deal with them in time. Therefore, oral health examination and education are effective means to prevent oral diseases. Through Oral Health Examination and Education programs, students can not only master oral epidemiology knowledge but also improve their oral examination skills and cultivate their concept of the public health service.

Although experiential service-learning is a powerful teaching method with many benefits for students, some potential shortcomings or challenges exist. Inequality of experiences and potential risks may be an obvious shortcoming of experiential service-learning teaching method [21]. Some students may have more access to opportunities for meaningful experiential service-learning due to their extroversion. In addition, experiential service-learning depends on external partners, such as communities, which can be another challenge if these partnerships are not well established or fail to satisfy the demands of the students. There are several shortcomings in the study. Firstly, the dental students did not perform the consistency check on site, which may affect the reliability of the caries data. Secondly, the questionnaire for three grades were completely consistent, which may be unreasonable. The children in the junior classes may not fully understand the meaning of some questions, such as “can I avoid dental caries by sealing the pits and fissures”. Thirdly, the project did not make any evaluation, so the effect of the improvement about the achievements with the intervention for the preschool children was unknown.

There are several suggestions for the Oral Health Examination and Education project. First, a major challenge was the communication with the community. The course had to be rescheduled several times, which inevitably affected students’ practical learning. Therefore, communicating with the community to confirm the visiting time before creating the schedule is necessary. Moreover, the establishment of multiple community partners and the preparation of alternatives before class can function as effective ways to deal with the problems of postponing classes. Another challenge was that the students were not skilled enough to check all the children’s oral cavities in a limited time. Additionally, according to the students’ feedback, they expected to receive preview materials before the theoretical class to deepen their understanding of the course and improve their efficiency. Therefore, preview materials such as videos and interactive tutorials on oral cavity examination should be distributed before the theoretical class. Furthermore, hands-on training should be provided before students go into the community, such as simulated clinical practice, with the aim of helping them develop necessary skills required in the oral examination and the confidence to perform oral health examinations on children.

## Conclusion

In conclusion, this study described an example of an experiential service-learning in preventive dentistry, which was implemented to meet the oral health care needs of a pediatric population. For dental students, the project not only exposed them to community service, but also benefit their understanding of the relevance and practical application of their coursework. Therefore, oral epidemiology instructors in oral and public health programs should consider incorporating community-based service-learning within their curricula to better prepare students for careers in oral public health.

## Data Availability

The datasets used and/or analyzed during the current study are available from the corresponding author on reasonable request.
