# Correction: An experiential service-learning project on oral health examination and education

**DOI:** 10.1186/s12909-024-05112-y

**Published:** 2024-02-14

**Authors:** Liangyue Pang, Yan Zhou, Ye Tao, Lixia Yu, Yina Cao, Huancai Lin, Qinghui Zhi

**Affiliations:** 1 Hospital of Stomatology, Sun Yat-sen University, Guangzhou, Guangdong China; 2 Guangdong Provincial Key Laboratory of Stomatology, Sun Yat-sen University, Guangzhou, Guangdong China

**Correction: BMC Medical Education (2024) 24:34**

10.1186/s12909-023-05020-7

Following publication of the original article [1], the authors informed us that the author Huancai Lin shares the corresponding authorship.

Also, the data in the third row of Table 1 should be “1.75 ± 1.83”, “1.46 ± 1.52” and “1.02 ± 1.12 ”. The symbol “_” is missing.

The original article has been corrected.
